# A Bottleneck Model of Set-Specific Capture

**DOI:** 10.1371/journal.pone.0088313

**Published:** 2014-02-07

**Authors:** Katherine Sledge Moore, Daniel H. Weissman

**Affiliations:** 1 Department of Psychology, Elmhurst College, Elmhurst, Illinois, United States of America; 2 Department of Psychology, University of Michigan, Ann Arbor, Michigan, United States of America; Goldsmiths, University of London, UK, United Kingdom

## Abstract

*Set-specific* contingent attentional capture is a particularly strong form of capture that occurs when multiple attentional sets guide visual search (e.g., “search for green letters” *and* “search for orange letters”). In this type of capture, a potential target that matches one attentional set (e.g. a green stimulus) impairs the ability to identify a temporally proximal target that matches another attentional set (e.g. an orange stimulus). In the present study, we investigated whether set-specific capture stems from a bottleneck in working memory or from a depletion of limited resources that are distributed across multiple attentional sets. In each trial, participants searched a rapid serial visual presentation (RSVP) stream for up to three target letters (T1–T3) that could appear in any of three target colors (orange, green, or lavender). The most revealing findings came from trials in which T1 and T2 matched different attentional sets and were both identified. In these trials, T3 accuracy was lower when it did not match T1’s set than when it did match, but only when participants failed to identify T2. These findings support a bottleneck model of set-specific capture in which a limited-capacity mechanism in working memory enhances only one attentional set at a time, rather than a resource model in which processing capacity is simultaneously distributed across multiple attentional sets.

## Introduction

Selective attention facilitates the processing of goal-relevant stimuli while inhibiting the processing of irrelevant stimuli. To accomplish this objective, it is thought that one or more attentional sets are created in memory, which define targets related to current goals using perceptual or conceptual attributes such as color, location, and semantic category [1]–[5]. Attentional sets then bias sensory systems to boost the signals of incoming stimuli that possess goal-relevant attributes, thereby enhancing the processing of those stimuli [6].

However, attentional sets also incur costs. For instance, in the contingent attentional capture paradigm, participants’ attention is involuntarily drawn to a distractor (e.g., a red digit) that closely resembles participants’ current attentional set for targets (e.g., search for red letters), thereby slowing or impairing subsequent target identification. In the classic cueing version of this paradigm, contingent attentional capture manifests as slowed reaction times to identify a target (e.g., a red letter) that appears in a different location than an immediately-preceding distractor that matches the target’s attentional set (e.g., a red digit) [4]. In a dynamic rapid serial visual presentation (RSVP) paradigm, capture is demonstrated as lower target identification accuracy when a target appears shortly following a distractor sharing features with the target’s atttentional set [7]–[10].

Several studies have further demonstrated that people are capable of maintaining more than one attentional set at a time while completing a search task [1], [2], [9]–[16], but there is an ongoing debate as to whether this incurs greater costs than maintaining a single set. Data from some studies suggest that multiple attentional sets can guide search without incurring additional costs [9], [12]. However, data from other studies suggest the opposite conclusion [13], [16].

Our initial investigation of this issue provided a potential explanation for this disagreement across studies [9]. In our experiments, participants viewed a rapid serial visual presentation (RSVP) stream at fixation that contained a series of colored letters, each appearing for about 100 ms. Two irrelevant RSVP streams–one to the left and one to the right–contained mostly grey items that appeared simultaneously with the letters in the central RSVP stream. Participants were asked to identify occasional targets in the central stream that appeared in particular target color(s).

In experiment 1, participants searched for letters appearing in one target color (i.e., by maintaining one attentional set) during half of the experiment and searched for letters appearing in two possible target colors (i.e., by maintaining two attentional sets) during the other half of the experiment. The chromatic environments were entirely different in order to minimize interference from one half of the experiment to the other. When no colored distractors appeared in the periphery during “active search”, we found no costs associated with maintaining two attentional sets relative to maintaining one attentional set, consistent with [12]. However, a major cost was incurred when maintaining two sets under a particular condition of distraction. More specifically, when a distractor that matched one attentional set (e.g. a green digit) appeared about 150–300 ms prior to a target that matched a different attentional set (e.g. an orange letter), target identification scores dropped by about 40%. This effect, which we called *set-specific capture*, was two to three times larger than when the distractor and target matched the same attentional set (i.e., traditionally-defined contingent attentional capture). Thus, it has been suggested that set-specific capture could be the source of switch costs in experiments that report an additional cost for maintaining more than one set at a time [13].

We concluded that set-specific capture is the result of a temporary, involuntary enhancement of one of the attentional sets being maintained, and ruled out several alternative explanations. More specifically, our view posits that detecting a potential target (e.g., a target-colored item) leads to a short-lived enhancement of the corresponding attentional set in working memory, which makes it harder to identify a subsequent potential target that matches a different set. Converging evidence for this explanation came from a second experiment in which distractors occasionally appeared just after the target, rather than slightly beforehand ( [9], Exp 3). Here, the set-specific capture effect reversed–accuracy was *higher* when the distractor and target matched different sets than when they matched the same set. We interpreted this finding as indicating that the initial target’s set was enhanced in working memory, thereby rendering a subsequent distractor less likely to be attended, and thus *less* disturbing, when it did not match the target’s set than when it did match.

A third experiment ruled out the possibility that set-specific capture is a manifestation of bottom-up perceptual priming of the target’s color ( [9], Exp 2). Here, participants were asked to search for letters in *any* color while ignoring grey letters, rather than to search for letters in either of two colors (e.g., orange and green) while ignoring letters in other colors. Critically, in the *any color* condition, there was no difference in capture magnitude when the target and distractor were the same versus different colors. In other words, the “set-specific” capture effect vanished when participants maintained a single set containing all colors. This result indicates that set-specific capture is not due to bottom-up perceptual priming.

**Figure 1:**
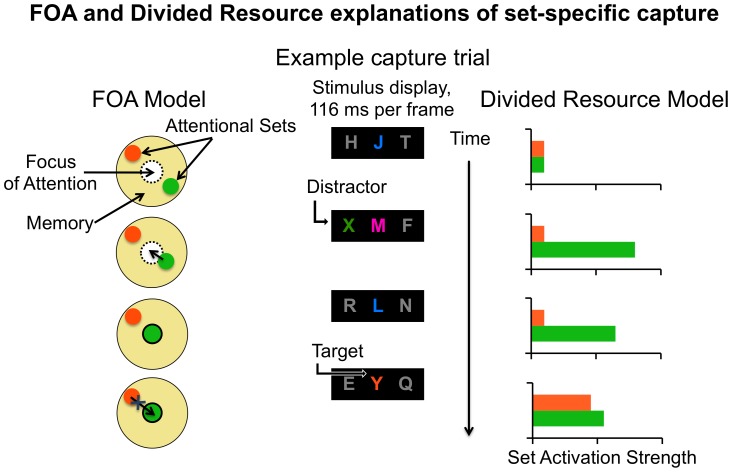
A schematic of the FOA and divided resource models indicating how they explain set-specific capture. In this example, participants are maintaining two attentional sets: “search for green letters in the central display” and “search for orange letters in the central display.” Peripheral displays contain distractors that are sometimes target-colored. Items in each of the three RSVP displays change identity every 166 ms. In this trial, a green distractor (X) matches one set and appears two frames prior to an orange target (Y). In the FOA model, depicted on the left, attentional sets reside in memory during active search. The focus of attention is empty until a target-colored item is detected, at which time the item’s corresponding set enters the focus. Only one set can be inside the focus at any given time. Thus, in this example, the orange target is not able to be identified because the green set still occupies the focus of attention when the orange target appears. In the divided resources model, depicted on the right, resources are divided unequally among concurrently-maintained attentional sets. Once a target-colored distractor is detected, its corresponding set is enhanced relative to other concurrently-maintained sets. When a target appears shortly afterward, its corresponding set is also enhanced. However, this enhancement is reduced as it stems from resources that are already partially allocated to enhancing the distractor’s set. In this example, target detection fails because too many resources were allocated to enhancing the distractor’s set when the target appeared.

Given the large size of set-specific capture effects, it is important to investigate which model best accounts for them. One possibility is a limited-resource model of attention, which posits a shared resource for concurrently maintained items or task rules [17], [18]. According to what we call the *divided resource model*, detecting a potential target (e.g., an orange distractor) leads the attentional system to allocate additional resources to the corresponding attentional set (e.g., “search for orange items”) in working memory. In this view, set-specific capture occurs because fewer resources are allocated to the attentional set that is needed to identify a subsequent differently-colored target (e.g., a green letter) than to the attentional set that is needed to identify a subsequent same-colored target (e.g., an orange letter). This view is consistent with prior work suggesting that attention can be divided unequally across multiple attentional sets [19]–[26]. Figure 1 (right) illustrates how the divided resources model explains the set-specific capture effect in our previous studies [9], [10].

A second model that could possibility explain set-specific capture, which we suggested in our previous investigations, posits that set-specific capture results from a bottleneck in working memory [9], [10]. This model, which we called the *focus of attention (FOA) model*, falls into the “bottleneck” class of models used to describe limitations of attention. In bottleneck models, only a single item can be processed during one or more cognitive stages [27]–[29]. The FOA model in particular is motivated by a theory of working memory in which a limited-capacity FOA can maintain only a single item or representation at any given time [30]–[32]. Evidence to support a FOA with a capacity of one item comes from the working memory [33], [34], task-switching [35], and attentional blink literatures [36], [37]. This view also fits with various models of selective attention. For example, according to coherence theory, focused attention can only be allocated to a single object at a time [38]. Similarly, a recent review of studies investigating the influence of working memory on selective attention concluded that only one attentional set can occupy the focus of attention at any given time [39].

According to the *FOA model* [9], [10], attentional sets are maintained in memory (but outside the FOA) during active search. From this vantage point, they facilitate the detection of potential targets (e.g., orange items and green items) by boosting the signals of incoming stimuli that possess target-defining attributes (e.g., orange and green features). When a potential target (e.g., a green distractor) is detected, the corresponding set (e.g., “search for green letters”) enters the FOA, which enables identification of the potential target. Critically, this set remains in the FOA at least until the potential target is identified. Set-specific capture therefore arises when the set (e.g., “search for orange letters”) needed to identify a subsequent differently-colored target (e.g., an orange letter) cannot enter the already-occupied FOA. Figure 1 (left) provides a schematic explanation of how the FOA model explains set-specific capture [9], [10].

In the present study, we sought to distinguish between the divided resource and FOA models of set-specific capture using a modified attentional blink paradigm. In a typical attentional blink experiment, participants are required to identify two or more targets within a single RSVP stream using the same attentional set (e.g. “identify the two digits that appear among the rapidly changing display of letters”). Typically, when the second target (T2) appears soon (but not immediately) after the first target (T1), T2 identification is impaired. In our modified attentional blink paradigm, participants searched for three targets that were defined by three different attentional sets, rather than just one set. Specifically, they searched for orange, green, and lavender targets in an RSVP stream containing heterogeneously-colored letters. In each trial, one to three targets appeared. Critical trials were those in which (a) three targets (T1–T3) appeared, (b) T1 and T2 were both identified, and (c) T1 (e.g., a green ‘H’) and T2 (e.g., an orange ‘G’) matched different attentional sets. In these trials, we determined whether T3 accuracy was higher when T3 (e.g., a green ‘T’) matched T1’s set than when T3 (e.g. a lavender ‘T’) matched neither T1’s nor T2’s set.

Crucially, the FOA and divided resource models make different predictions about relative T3 identification accuracy in these two trial types. The FOA model predicts that T3 accuracy should be uniformly poor in these trial types because only T2’s set occupies the focus of attention and T3 does not match T2’s set for either trial type. In contrast, the divided resource model predicts higher T3 accuracy when T3 matches T1’s set than when it does not. According to this model, resources are allocated primarily to T2’s set after T2 has been identified, but some resources are also still allocated to T1’s set. Thus, T3 should be identified more accurately when it matches either of these sets – even T1’s – than when it matches neither of them. As described next, our findings provide novel support for the FOA model.

## Methods

### Ethical Statement

This study was approved by the University of Michigan Behavioral Sciences Institutional Review Board. Participants provided written informed consent before starting the experiment.

### Participants

Forty-two University of Michigan students aged 18 to 25 years participated in exchange for course credit. All reported normal or corrected vision.

### Apparatus and Stimuli

Colored letter stimuli were displayed on a 19″ Viewsonic CRT monitor with a 60 Hz refresh rate, controlled by a PC running Windows XP. Presentation software (Neurobehavioral Systems, Inc.) displayed the stimuli and collected responses. A viewing distance of 80 cm was enforced by a chin rest.

In each trial, fifteen colored letters (2.07°×1.88° in size) appeared successively (duration, 150 ms; inter-stimulus gap, 16 ms) in a central RSVP stream overlaid on a black background. We used all possible letters except “I”, “O”, and “W”. No letter was repeated within a trial.

**Figure 2:**
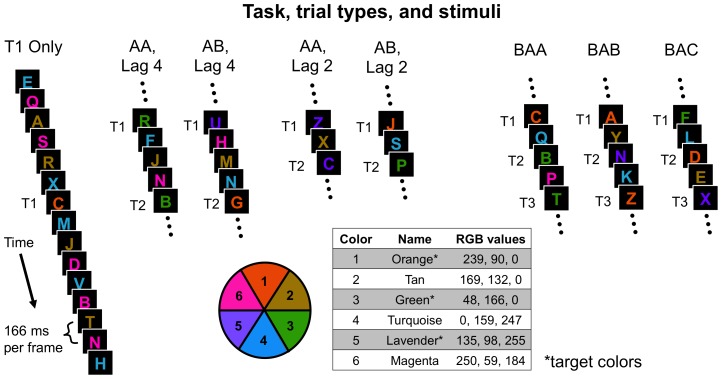
Task, trial types, and stimuli. In each trial, participants viewed 15 colored letters in rapid succession. One to three of these letters were targets defined by color (orange, green, or lavender) that participants were supposed to identify at the end of the trial. Non-target colors were tan, magenta, and turquoise. Each letter appeared for 150 ms followed by a blank space lasting 16 ms. In the figure, the T1 Only trial type is illustrated using a full sequence of 15 letters. The other trial types are illustrated more briefly by showing only targets and intervening non-targets. The first target (T1) appeared randomly between the fourth and eighth positions in the RSVP stream (inclusive). AA and AB trials contained two targets (T1 & T2) that were the same (AA) or different (AB) colors. T1 and T2 were separated by one (lag 2) or three (lag 4) intervening items. BAA, BAB, and BAC trials contained three targets (T1, T2 & T3), each of which was separated by one intervening item. In BAA trials, T2 and T3 were the same color while T1 was a different color. In BAB trials, T1 and T3 were the same color while T2 was a different color. In BAC trials, T1, T2, and T3 were all different colors.

We colored the letters according to the “light” scheme of Moore & Weissman, 2010 [9]. Target letters were colored orange, lavender, and green. Non-target letters were colored magenta, tan, and turquoise (see Figure 2 for a color wheel and for “RGB” values). This color scheme ensured near-uniform salience and luminance of all stimuli, which is important because salience can contribute to capture effects [40], [41]. A control experiment verified that the target colors were equally discriminable from each other (see [9]). This color scheme also ensured that a distractor color separated each of the target colors in color space [42]. Thus, participants could only perform the task by maintaining three independent attentional sets for color [9], [10], [14]. Adjacent non-target letters in the stream were always differently colored.

### Procedure

Participants were instructed to identify any letters in each 15-item trial whose color matched one of the three target colors (orange, green, and lavender). They were also told that one, two, or three targets could appear in each trial. T1 appeared randomly between the fourth and eighth positions (inclusive) in the RSVP stream.

At the end of each trial, three prompts appeared successively on the computer screen. The first stated “Please type the first target letter.” After recording a response, analogous second and third prompts asked participants to identify the second and third targets. All three prompts were presented on every trial, even when just one or two targets were presented. We reasoned that uncertainty about the number of targets in each trial would lead to relatively few false alarms (i.e., guesses). Participants were told to press the space bar in response to any prompt for which they did not perceive a target (e.g., to press the spacebar for the third prompt if only two targets were perceived). There was no pressure to respond quickly. The next trial began 500 ms after participants responded to the third prompt.

### Design

There were eight trial types (see Figure 2). In T1 Only trials (n = 168), a single target appeared. The remaining seven trial types are labeled using two- and three-letter sequences, in which “A,” “B,” and “C” are variables that represent different target colors. Two trial types contained two targets. In AA trials (n = 84), T1 and T2 were the same color, while in AB trials (n = 84) T1 and T2 were different colors. For half of AA and half of AB trials, T1 and T2 were separated by one intervening non-target item (Lag 2). In the other half, T1 and T2 were separated by three intervening non-target items (Lag 4).

Five trial types contained three targets: BAA, BAB, BAC, AAA, and AAB. In these trial types, the targets were always separated by one intervening non-target item. There were 42 trials in each of the BAA, BAB, and BAC trial types. In these critical conditions, T1 and T2 were always different colors. T3’s color matched T1’s color (BAB), T2’s color (BAA), or neither (BAC). AAA and AAB trials (not pictured) were not critical for distinguishing between the FOA and divided resource hypotheses. However, we included them in smaller numbers (n = 24 each) to ensure that (1) participants would not develop expectations about T3’s color based on T1’s color and T2’s color and (2) an equal number of trials (168) contained one, two, and three targets. Because these trial types were not crucial for testing our hypotheses, they will not be further discussed. Finally, for each of these trial types, all possible color combinations were represented in equal numbers.

### Data Analysis

Accuracy (percentage of hits) was the dependent measure in all analyses. Some analyses (e.g., T3 accuracy) were contingent upon whether participants identified *or missed* certain targets within a given trial (e.g., T1 and/or T2). We considered misses and incorrect responses (i.e. false alarms) for the purposes of these analyses. A separate calculation found that just 8.4% of trials were false alarms; the remaining were misses. Because some analyses were dependent on whether a prior target had been identified, there were often unequal numbers of trials per condition. We included only those participants with at least four trials in *each* of the following critical conditions: T1 identified in AA and AB trials (four or more trials at each lag); T1 missed in AA and AB trials (four or more trials at each lag); both T1 and T2 identified in BAA, BAB, and BAC trials; and T1 identified but T2 missed in BAA, BAB, and BAC trials (see Table 1 for more detailed information regarding trial counts). We eliminated two participants for poor performance (e.g., too few trials in a “difficult” condition, such as T1 and T2 correct in BAC trials) and five participants for excellent performance (e.g., too few trials in an “easy” condition requiring a T1 or T2 miss, such as T1 missed in AA trials.) Altogether, we eliminated seven participants, leaving thirty-five in the final analyses.

**Table 1:** Trial counts for each condition.

	Avg N trials	range
AA Lag 2 | T1	35.3	33–38
AB Lag 2 | T1	34.8	32–37
AA Lag 4 | T1	34.5	30–38
AB Lag 4 | T1	34.9	34–38
AA Lag 2 | no T1	6.7	4–9
AB Lag 2 | no T1	7.2	5–10
AA Lag 4 | no T1	7.5	4–12
AB Lag 4 | no T1	7.1	4–8
BAA | T1, T2	16.38	5–22
BAB | T1, T2	15.96	4–24
BAC | T1, T2	15.54	7–21
BAA | T1, no T2	18.42	9–25
BAB | T1, no T2	19.24	8–29
BAC | T1, no T2	18.86	7–31

Column 1 indicates condition name. Column 2 indicates the average number of trials per condition across participants. Column 3 indicates the range of trials for each condition across participants.

As stated earlier, our accuracy-based analyses led different participants to have different numbers of trials per condition. We therefore used the conservative Welch t-test, as this statistic does not require different trial types to have equal variance. Notably, this t-test calculates the degrees of freedom based on the estimated variance in each trial type, and so the degrees of freedom change across analyses. Note that we repeated all of our analyses using conventional t-test statistics as well, and obtained the same results. We also verified that the most important findings involving two- and three-target trials were observed with repeated-measure analyses of variance (ANOVA). However, only the Welch t-test results will be reported here because our data do not fully conform to the assumptions required of the other analysis methods.

## Results

**Figure 3:**
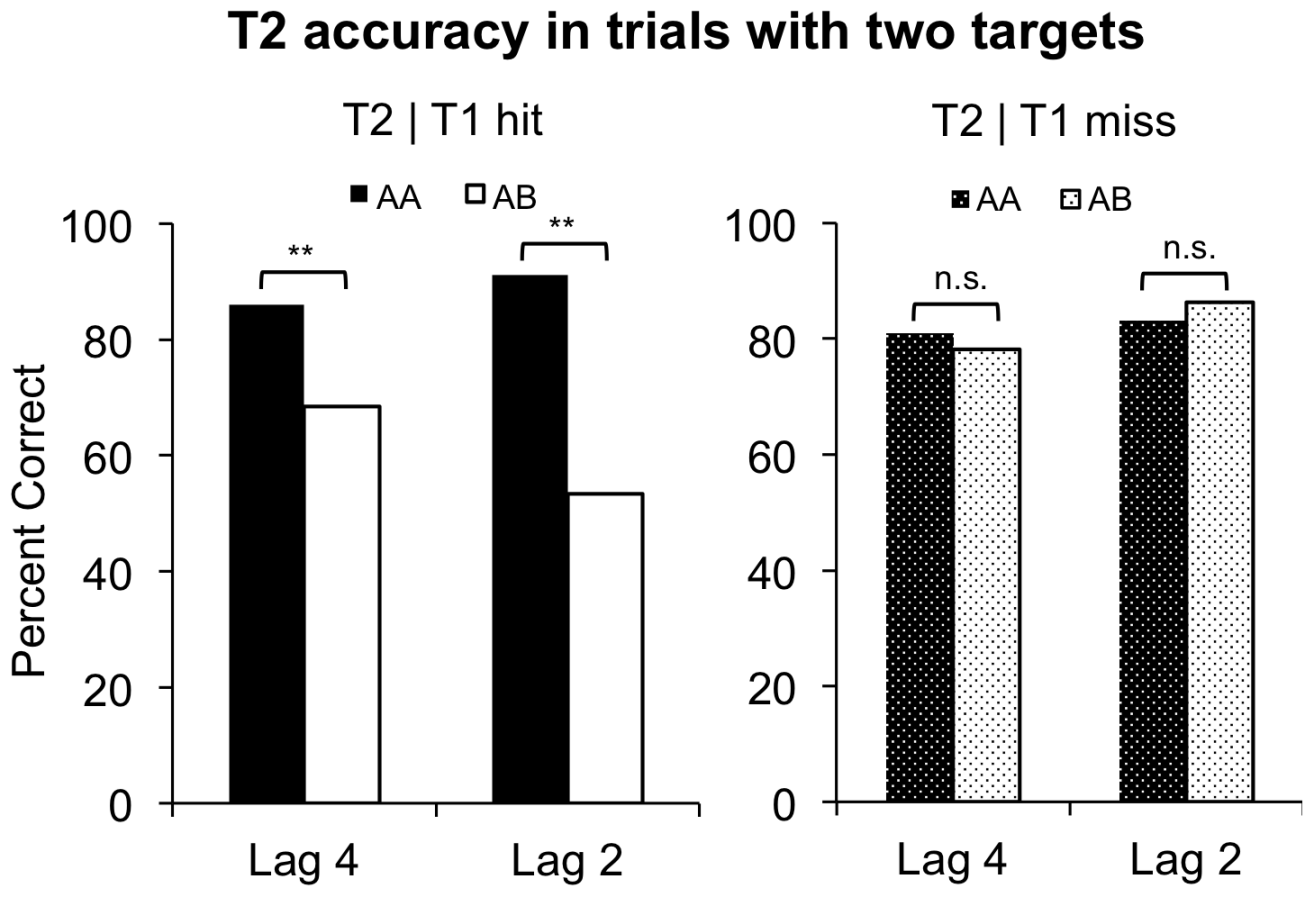
T2 accuracy in trials with two targets. The left panel plots T2 accuracy given that T1 was identified. Consistent with our prior findings of set-specific capture, T2 accuracy was lower in AB than in AA trials at both Lag 2 and Lag 4. A double asterisk indicates a p-value less than 0.0005. The right panel plots T2 accuracy given that T1 was missed or reported incorrectly. In these trials, T2 accuracy did not vary with T1’s color.

**Figure 4:**
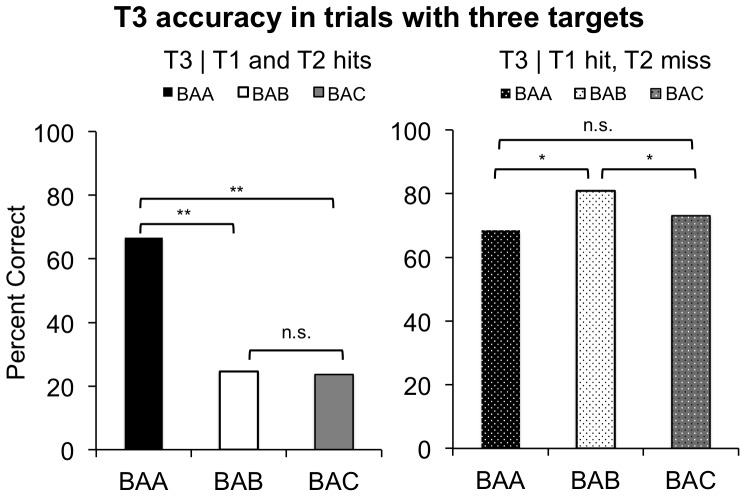
T3 accuracy in trials with three targets. The left panel shows T3 accuracy in BAA, BAB, and BAC trials given that both T1 and T2 were identified. Consistent with set-specific capture, T3 accuracy was lower when T2 and T3 matched different attentional sets (BAB and BAC trials) than when they matched the same attentional set (BAA trials). Critically, in line with the FOA model, T3 accuracy did not vary with whether T1 and T3 matched the same attentional set: T3 accuracy in BAB and BAC trials did not significantly differ. The right panel shows T3 accuracy given that T1 was identified and T2 was missed. In line with the FOA model, in these trials T1’s attentional set *did* influence T3 accuracy, while T2’s set did not. That is, T3 accuracy was higher in BAB trials than in either BAA or BAC trials, but did not differ between BAA and BAC trials. A double asterisk indicates p<0.0005, and a single asterisk indicates p<0.05.

We began by verifying the presence of set-specific capture in our task. As expected, when T1 was identified in two-target trials, T2 accuracy was lower when T1 and T2 matched different attentional sets than when they matched the same set. That is, performance was worse in AB than in AA trials at both lag 2 [AB = 53.4%, AA = 91.2%, t(59) = 7.07, p<0.0001] and lag 4 [AB = 68.4%, AA = 86.0%, t(48) = 4.78, p<0.0001] (Figure 3, left) Also consistent with previous findings, set-specific capture was less pronounced at lag 4 than at lag 2 [F(1,35) = 14.8, p<0.0001], indicating that participants recover from such capture at longer SOAs. Set-specific capture was also present in three-target trials when both T1 and T2 were identified: T3 accuracy was lower when T2 and T3 matched different sets than when they matched the same set. More concretely, performance was worse in BAB (24.6%) than in BAA (66.6%) trials [t(67) = 6.78, p<0.0001] and worse in BAC (23.6%) than in BAA trials [t(69) = 6.83, p<0.0001] (Figure 4, left).

**Figure 5:**
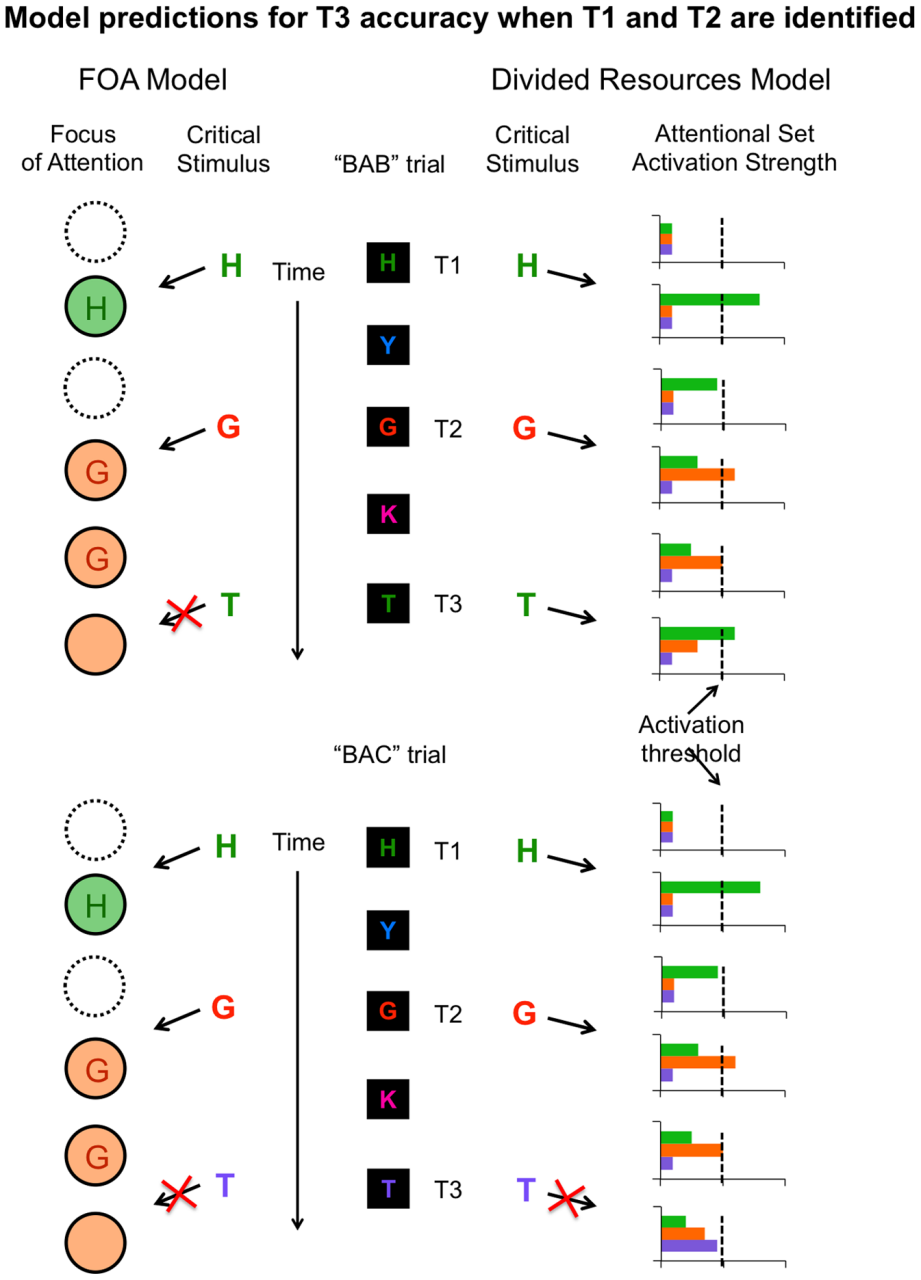
A comparison of the predictions made by the FOA and divided resource models for T3 accuracy in BAB vs. BAC trials when T1 and T2 are both identified. In the FOA model (left), identifying a target letter requires the corresponding attentional set to be enhanced inside the FOA. In each trial, the focus is empty prior to the appearance of the first target, as indicated by the circle with the dotted line. If a target is identified, the corresponding set enters and occupies the FOA, as indicated by the change in color of the FOA. No other set can be enhanced at the same time, as indicated by a solid line around the focus. Given these assumptions, the FOA model predicts that T3 is equally unlikely to be identified in BAB and BAC trials because T2’s set already occupies the FOA when T3 appears. In the divided resources model (right), an attentional set can be activated in a graded manner. In order for a target to be identified, its corresponding set must be activated above a certain threshold level, as depicted by the dotted line. Critically, the divided resources model predicts that T3 is more likely to be identified in BAB than in BAC trials because, in BAB trials, there is lingering enhancement of T1’s set even after T2’s set has been activated and T2 has been identified. This lingering enhancement should make it relatively easy for T3’s set (which matches T1’s set) to reach the activation threshold for identification. In BAC trials, there is no lingering enhancement of T1’s set, which should make it relatively hard for T3’s set to reach the activation threshold.

Having verified the presence of set-specific capture in our task, we sought to distinguish between the FOA and divided resource models. Specifically, we contrasted T3 accuracy in BAB and BAC trials when both T1 and T2 were identified. Figure 5 provides a schematic of how the FOA and divided resource models predict different outcomes for BAB and BAC trials. As uniquely predicted by the FOA model, T3 accuracy did not differ in BAB and BAC trials [t(67) = 0.046, p = 0.963] (Figure 4, left). That is, inconsistent with the divided resource model, we observed no evidence that an initial enhancement of T1’s set had a positive impact on subsequent T3 identification in BAB trials when T2 was also identified.

What if enhancement of T1’s set was present during T2 identification, but dissipated before T3 appeared? If this was the case, then the divided resources model would still be able to explain the pattern of results observed above. To explore this possibility, we contrasted T3 accuracy in BAC, BAB, and BAA trials when T1 was identified and T2 was missed. We reasoned that if enhancement of T1’s set wore off before T3 appeared, then T3 accuracy would not be higher in BAB trials than in the other trial types. Contrary to this prediction, T3 accuracy was higher in BAB (80.9%) than in both BAC (73.1%) [t(65) = 2.30, p<0.025] and BAA (68.4%) [t(54) = 2.72, p<0.008] trials (Figure 4, right). Thus, enhancement of T1’s set lasted until T3 appeared, but only when T2 was *not* identified. This result suggests that enhancement of T1’s set needed to be terminated for the system to enhance T2’s set and identify T2. It therefore supports the FOA model’s claim that only one set can occupy the FOA.

We have argued that T1’s attentional set exerts no influence on T3 identification accuracy when T2 is identified. If this is the case, however, then why was T3 accuracy in three-target trials lower than T2 accuracy in lag 2, two-target trials when T1 and T3 matched different sets and all previous targets were identified? More specifically, why was (a) T3 accuracy in BAA trials lower than T2 accuracy in lag 2, AA trials and (b) T3 accuracy in BAC trials lower than T2 accuracy in lag 2, AB trials when all previous targets were identified (both ps <0.001)? According to the FOA model, the accuracy with which the final target is identified in these trial types should not differ because it should depend only on the most recent, previously-identified target’s set.

One possible interpretation, which is inconsistent with the FOA model, is that this effect indexes a lingering influence of T1’s set in three-target trials that influences T3 accuracy even when T2 is identified. However, this interpretation is at odds with our findings above indicating equivalent performance in BAB and BAC trials when T1 and T2 were both identified. Indeed, if enhancement of T1’s set lingered to influence T3 performance, then T3 accuracy should have been higher in BAB trials than in BAC trials. In addition, lingering enhancement of T1’s set predicts T3 accuracy in BAB trials to be at least as high as (if not higher than) T2 accuracy in lag 2, AB trials; however, we observed the opposite result, t(51) = 11.1, p<0.0001.

A second possible interpretation, which is also inconsistent with the FOA model, is that this effect indexes a performance trade-off between T1 accuracy and the accuracy with which the final target is identified, consistent with resource depletion accounts of target identification accuracy in RSVP tasks [43]. Contrary to this view, however, a comprehensive series of pairwise comparisons indicated that T1 accuracy did not differ between trial types (p>0.1 for all comparisons). These findings weigh against a performance trade-off between T1 accuracy and the accuracy with which the final target was identified. However, they appear consistent with the FOA model, which posits that set-specific capture occurs only when the FOA is occupied by the set corresponding to a previously-identified potential target. Indeed, in most situations, the FOA should not be occupied when T1 appears.

A third possible interpretation, which is *not* inconsistent with the FOA model, is that this effect indexes greater working memory demands on three-target trials than on two-target trials, which result in reduced T3 accuracy. Indeed, when all previous targets are identified, three responses need to be maintained in three-target trials while only two must be maintained in two-target trials. To investigate this possibility, we determined whether the difference between T3 accuracy in three-target trials and T2 accuracy in two-target trials mentioned earlier was no longer present in three- and two-target trials that imposed equivalent working memory demands. More specifically, we compared (a) T3 accuracy in three-target trials (BAA, BAB, BAC) wherein T1 was identified and T2 was missed (Figure 4, right) to (b) T2 accuracy in lag 4, two-target trials (AA, AB) wherein T1 was identified (Figure 3, left). In each of these trial types, participants maintained one response prior to the appearance of the critical target. Further, the first and last targets were separated by a lag of 4. Thus, the working memory demands imposed by these trial types were closely matched. To preclude the possibility that differences in set-specific capture might lead to spurious differences between T3 and T2 accuracy for these memory-equated trial types, we only compared trial types in which the two critical targets both matched the same set or both matched different sets.

Consistent with a working memory explanation, we observed no differences between T3 accuracy and T2 accuracy for the memory-equated trial types above. First, T3 accuracy in BAB trials did not differ from T2 accuracy in AA trials [t(67) = 1.65, p = 0.104]. Second, T3 accuracy in BAA trials did not differ from T2 accuracy in AB trials [t(62) = 0.013, p = 0.990]. Third, T3 accuracy in BAC trials did not differ from T2 accuracy in AB trials [t(67) = 1.13, p = 0.264]. These findings suggest that when all previous targets were identified, differences between T3 accuracy in three-target trials and T2 accuracy in two-target trials were likely driven by the unequal working memory demands these trial types imposed.

Consistent with the FOA model, the findings above also suggest that missed targets do not give rise to set-specific capture. Two additional results confirmed this hypothesis. First, in two-target trials, set-specific capture was absent when T1 was missed: T2 accuracy did not differ in AA and AB trials at lag 2 [AA = 83.1%, AB = 86.3%, t(56) = 0.745, p = 0.459] and at lag 4 [AA = 80.8%, AB = 78.2%, t(59) = 0.691, p = 0.492] (Figure 3, right). Second, in three-target trials, set-specific capture arising from T2 was absent when T1 was identified and T2 was missed: T3 accuracy did not differ in BAA and BAC trials [t(61) = 0.949, p = 0.346] (Figure 4, right). These findings fit with the FOA model’s assumption that a potential target is often missed because its corresponding attentional set fails to enter the FOA [9], [10]. They also provide converging evidence against trivial explanations of set-specific capture that involve bottom-up perceptual priming of an upcoming target’s color [9].

## Discussion

We employed an RSVP task to investigate whether *set-specific* capture is better explained by a bottleneck in working memory (the FOA model) or by a depletion of resources that are distributed across multiple attentional sets (the divided resources model). We found that when T1 and T2 matched different sets and were *both* identified, T3 identification accuracy was no greater when T3 matched T1’s set than when T3 matched neither T1’s nor T2’s set. Critically, this result appears more consistent with the FOA model than with the divided resources model.

Could a lingering enhancement of T1’s set, present when T2 was identified, have worn off before T3 appeared? If so, then the divided resources model would still be able to account for the present results. This possibility appears unlikely, however, for the following reason: when T1 was identified but T2 was missed (and, hence T2’s set was not enhanced), T3 performance was better when T3 matched T1’s set than when it did not. This result suggests that enhancement of T1’s set did not wear off before T3 appeared unless such enhancement was terminated by the enhancement of T2’s set. Thus, in line with the FOA model, it would appear that the enhancement of T1’s set was terminated abruptly when T2 was identified, rather than gradually as would be predicted by the divided resources model.

### How is Set-specific Capture Related to the Attentional Blink Phenomenon?

Given the similarity of our task to a typical attentional blink task, it is worth considering the relationship between *set-specific* capture and the attentional blink phenomenon. Traditional contingent attentional capture has been compared to the attentional blink phenomenon, and the consensus is that the same attentional limitations give rise to both effects [8], [44], [45]. However, set-specific capture and the attentional blink have not yet been compared, likely because attentional blink experiments typically involve maintaining just one attentional set at a time (e.g., search for letters within an RSVP stream containing mostly digits) [36]. Indeed, even when T1 and T2 *do* match different sets, they typically do so predictably (e.g., T1 is always a black letter and T2 is always a red digit) [36]. Thus, only one set needs to be maintained at any time. Models of the attentional blink therefore do not typically address the issue of maintaining multiple attentional sets.

However, similar to the FOA model of set-specific capture, at least one model of the attentional blink phenomenon - Boost and Bounce theory - posits it is possible to focus attention on only one attentional set at a time [46]. In line with this view, when participants switch between attentional sets in the attentional blink paradigm, as described earlier, T2 performance suffers. Specifically, Lag-1 sparing, wherein T2 identification is “spared” if T2 immediately follows T1 with no intervening items, is absent when T1 and T2 match different sets [47]. Future experiments might therefore investigate whether the FOA model explains not only set-specific capture, but also set switch costs in the attentional blink paradigm when multiple sets are maintained.

### Does Set-specific Capture Index a Conventional Task Switch?

The potential link between set-specific capture and switch costs in the attentional blink paradigm noted above raises the more general possibility that set-specific capture reflects a switch cost similar to those observed in conventional task switching studies [35]. At first blush, set-specific capture appears similar to task switching, which may also require replacing the attentional set, or task goal, that currently occupies the FOA with a different set or goal [35]. However, the processes underlying set-specific capture appear to differ from those underlying task switching in at least one important way. In task-switching studies, switch costs are exacerbated when participants switch back to a task they recently performed, suggesting that switching away from a task involves inhibiting its associated task set [48]. In contrast, such *backward task-set inhibition* does not characterize set-specific capture. Indeed, when both T1 and T2 were identified in the present study, T3 accuracy was no worse in BAB trials (where identifying T3 involved switching back to T1’s set) than in BAC trials (where identifying T3 did not involve switching back to T1’s set). Future studies might investigate whether this difference between set-specific capture and conventional task switching costs stems from the fact that these effects are measured over relatively short (set-specific capture) versus relatively long (task switching) timescales. Whatever the outcome, set-specific capture appears to differ from a conventional switch cost in at least one respect.

### Does Set-specific Capture Stem from an Immutable Bottleneck in Information Processing?

More broadly, it is important to consider whether the bottleneck in information processing that is implied by the FOA model is an immutable aspect of the cognitive architecture or a more flexible aspect that can be influenced by task strategies. Consistent with the latter possibility, some cognitive phenomena that are thought to stem from bottlenecks in information processing are highly sensitive to task strategies. For example, the attentional blink phenomenon, which results from a bottleneck according to some models [27], [28], [36], [49], [50], is reduced when participants adopt a strategy of “zoning out” or when they divide attention between the AB task and a different task [39], [51]–[53].

As another example of a more “flexible” bottleneck, consider the psychological refractory period (PRP), which refers to the delay in making the second of two choice reaction time responses when two targets (T1 and T2) appear in rapid succession [28]. The PRP effect was initially attributed to an immutable bottleneck wherein response selection for T2 could not begin until it was completed for T1 [28], [50], [54], [55]. Subsequent work, however, revealed that when participants were instructed to complete both tasks at the same time (rather than to prioritize the first task over the second as they are typically instructed), the PRP effect was eliminated, suggesting there is no immutable response selection bottleneck [56], [57]. Given such findings, future studies might investigate whether the bottleneck that gives rise to set-specific capture is also a flexible, rather than immutable, aspect of the information processing architecture.

### Caveats and Limitations

Although the present data appear most consistent with the FOA model, they do not completely rule out the divided resources model. For example, the divided resources model could explain our findings if resources were allocated in an all-or-none fashion to attentional sets, thereby mimicking an information processing bottleneck. A graded assignment of resources could also potentially explain our findings but only if some complex assumptions are made. First, the resources assigned to T1 would need to dissipate too quickly to observe a difference in T3 accuracy between BAB and BAC trials when T1 and T2 are both identified. Second, those same resources would need to linger long enough to observe such a difference when T2 is missed [58], [59]. Given that the present study was not designed to assess these complex assumptions, future studies will be needed to further test the divided resource model.

## Conclusion

The present findings appear more consistent with a bottleneck model of set-specific capture than with a resource model. Specifically, they confirm several predictions of the FOA model, which posits that a limited-capacity focus of attention in working memory can maintain only a single attentional set at any given time. They also differentiate set-specific capture from the attentional blink phenomenon, task switching, and from other forms of capture in which an uneven distribution of resources across multiple attentional sets determines performance.
